# ICP-MS-based quantitative analysis and risk assessment of metal(loid)s in fish species from Chennai, India

**DOI:** 10.3389/fpubh.2025.1609067

**Published:** 2025-07-08

**Authors:** Suryapratap Ray, Gracy Anu Francis, Sumit Sudhir Pathak, Pooja Chavan, Rahul Vashishth

**Affiliations:** 1 Department of Biosciences, School of Biosciences and Technology, Vellore Institute of Technology, Vellore, India; 2 Division of Food Processing Technology, Karunya Institute of Technology and Sciences, Coimbatore, Tamil Nadu, India; 3 VIT School of Agricultural Innovation and Advanced Learning, Vellore Institute of Technology, Vellore, India

**Keywords:** trace elements, environmental impacts, health risk assessment, sea food, contaminated fish

## Abstract

**Introduction:**

Presence of heavy metal pollutants indicates an alarming situation that disrupts marine trophic dynamics, presenting substantial threats to fish populations and ultimately affecting human societies that depend on these aquatic resources for sustainable nutrition.

**Methods:**

The present study focused on three fish species from Chennai (Tamil Nadu, India), namely *Nemipterus japonicus, Oreochromis mossambicus*, and *Lates calcarifer*. Heavy metal profiling was conducted on organs such as liver, gills, and muscle tissue. ICP-MS was utilized to determine the concentrations of heavy metals.

**Results:**

Upon analysis of Arsenic (As), Cadmium (Cd), Chromium (Cr), Mercury (Hg), Lead (Pb), Strontium (Sr), and Vanadium (V), the concentration ranges (dry weight) were observed as 0.044–0.096 μg/kg, 0.696–0.778 μg/kg, 5.259–12.399 μg/kg, 0.020–0.660 μg/kg, 15.400–17.649 μg/kg, 1.068–15.200 μg/kg, and 0.150–1.208 μg/kg, respectively, across the three fish species. The muscle tissues of *Oreochromis mossambicus* exhibited the highest heavy metal contamination, particularly due to its elevated Chromium (Cr) concentration of 12.399 μg/kg.

**Discussion:**

*Oreochromis mossambicus* recorded the highest Hazard Index (HI) in both children (0.238) and adults (0.136). However, the HQ and HI values were < 1, suggesting that consumption of these fish species remains within a safe limit regarding heavy metal contamination. These findings underscore the need for strict monitoring and regulatory measures to reduce further heavy metal contamination in seafood.

## 1 Introduction

Aquatic organisms, particularly fish, are highly susceptible to heavy metal contamination through direct exposure to polluted water and sediment (1). The bioaccumulation of these metals in fish can lead to serious health issues, including impaired reproductive capacity, reduced survival rates, and increased risks of cancer, birth defects, and genetic mutations (2, 3). Consequently, human populations that rely on fish as a dietary staple face potential health risk, including neurological disorders, organ damage, and increased susceptibility to chronic diseases (4). The increasing consumption of fish globally, with India's per capita fish intake ranging from 5 to 6 kg annually in the general population to 8–9 kg among fish-eating communities, further underscores the need for continuous monitoring of heavy metal contamination [(5, 6)].

Heavy metals are characterized by their high density and atomic weight, typically five times that of water. They include transition metals, metalloids, lanthanides, and actinides, many of which exhibit toxic effects even at trace levels (7). Among the most hazardous are Arsenic (As), Cadmium (Cd) Chromium (Cr), Mercury (Hg), Lead (Pb), Strontium (Sr) and Vanadium (V), originating from both natural and anthropogenic sources. These metals enter aquatic ecosystems through industrial effluents, agricultural runoff, fossil fuel combustion, and waste disposal (8–10). Once introduced, they accumulate in sediments and water, becoming bioavailable to aquatic organisms. Fish, as top consumers in the aquatic food web, absorb and retain these metals in their tissues, posing potential health risks to consumers (11, 12). Prolonged exposure to heavy metals has been associated with neurological disorders, cardiovascular diseases, kidney damage, and carcinogenic effects, making their presence in seafood a major public health issue (11, 13–15).

Previous studies have reported elevated heavy metal concentrations in fish from various regions of India, with levels often exceeding recommended safety thresholds. In particular, marine fish species from the Chennai coast, such as *Nemipterus japonicus* and *Rastrelliger kanagurta*, have shown high levels of arsenic, chromium, cadmium, and lead, especially in liver tissues (16). Although extensive research exists on heavy metal contamination in fish from temperate regions, data from tropical areas, particularly along India's southeast coast, remain comparatively limited. The Chennai coast, especially the Ennore region, has witnessed significant industrialization, resulting in elevated heavy metal discharge through industrial and domestic effluents. Over the past two decades, this region has experienced rapid urbanization and expansion of maritime activities (17–20). The region's escalating industrial activity, coupled with the extensive dependence of local communities on fisheries for both sustenance and livelihood, necessitates an urgent assessment of heavy metal accumulation in commercially important fish species. Understanding the extent of contamination and assessing the associated health risks are crucial for developing regulatory measures to ensure food safety.

This study aims to quantitatively evaluate heavy metal accumulation in fish species commonly consumed along the Chennai coast, particularly near Ennore—a region heavily impacted by rapid industrialization and urban effluent discharge. Inductively Coupled Plasma Mass Spectrometry (ICP-MS) was employed due to its high sensitivity, precision, and ability to detect trace levels of multiple metals simultaneously, making it ideal for environmental and food safety applications. To better understand the bioaccumulation patterns and public health implications, three specific fish tissues—liver, gills, and muscle—were selected for analysis. The liver is a primary detoxification organ and often reflects chronic metal exposure; gills are directly exposed to waterborne contaminants and represent respiratory uptake; muscle tissue, being the most commonly consumed part, directly indicates human dietary exposure. Seven metal(loid)s— Arsenic (As), Cadmium (Cd) Chromium (Cr), Mercury (Hg), Lead (Pb), Strontium (Sr) and Vanadium (V) were selected based on their high toxicity, persistence in the environment, and known bioaccumulation potential. These five metal(loid)s were selected based on their recognized toxicity, environmental persistence, and classification as priority pollutants or carcinogens by international agencies such as the WHO, USEPA and EFSA. Additionally, dietary risk assessment indices such as Estimated Daily Intake (EDI), Target Hazard Quotient (THQ), and Hazard Index (HI) were applied to evaluate potential health risks to local fish consumers. By integrating advanced analytical techniques with tissue-specific metal profiling and human health risk assessment, this study provides critical insights for environmental monitoring, regulatory action, and public health protection. The findings are expected to contribute valuable baseline data for food safety regulations and long-term ecological assessments in the southeastern coastal region of India.

## 2 Materials and method

### 2.1 Sampling location

**Figure 1:**
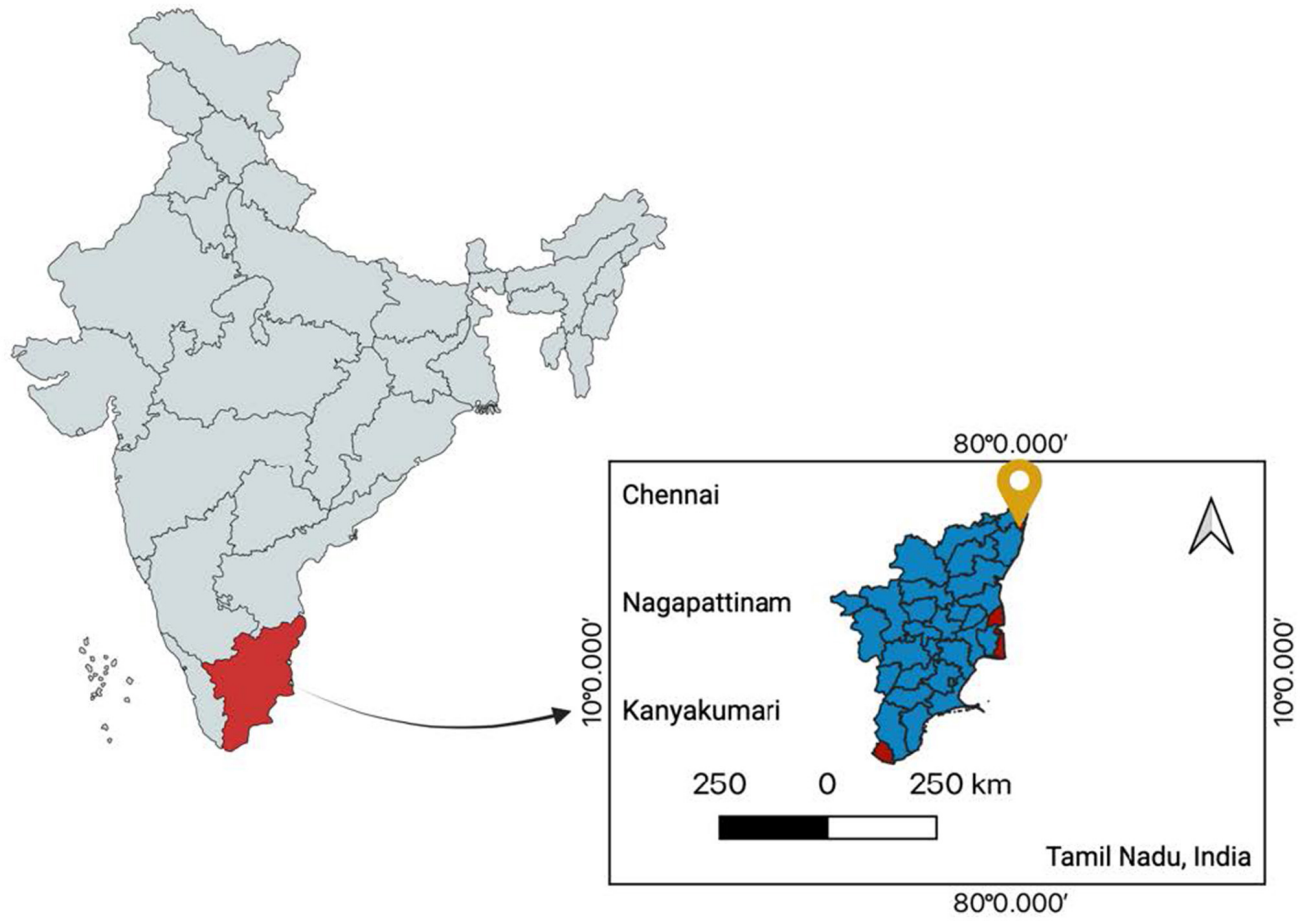
Location map of the sample collection site (Chennai, India) (13.0843°N, 80.2705°E) (Created in BioRender. Ray, S. (2025) https://BioRender.com/undefined).

The study was conducted in Ennore, Tamil Nadu, India (13.216°N, 80.324°E), a coastal ecosystem influenced by both natural and anthropogenic activities (Figure 1). This location was chosen due to its high fishery productivity and potential bioaccumulation of heavy metals, posing risks to both marine biodiversity and human health. Ennore is a major fishing hub but is highly impacted by industrial discharges, thermal power plants, petrochemical industries, and urban wastewater runoff (21). The Ennore Creek and adjacent coastal waters experience heavy metal contamination, affecting both marine biodiversity and public health.

### 2.2 Processing of samples

Fish samples were collected from a major marketplace in Chennai, Tamil Nadu, India (13.0843°N, 80.2705°E). Three commercially significant fish species, *Nemipterus japonicus, Oreochromis mossambicus*, and *Lates niloticus*, were selected due to their high local consumption rates and ecological significance (22). Vendor interviews and sourcing information indicated that the fish originated from coastal and inland waters surrounding Chennai. These aquatic environments are located near industrial zones, sewage outlets, and areas of agricultural runoff known sources of heavy metal contamination. Previous studies have identified elevated levels of toxic metals such as lead (Pb), cadmium (Cd), and mercury (Hg) in the Chennai coastal region, suggesting a potential pathway for bioaccumulation in fish species harvested from this water (17, 19). A total of 18 fish specimens were collected by adopting a purposive randomized sampling technique, with six individuals representing each of the three species (23). This number was selected based on species availability at the time of collection, and the resource-intensive nature of ICP-MS analysis. While this sample size allows for preliminary insights into species-specific heavy metal accumulation, it is recognized as a limiting factor for drawing broader statistical inferences. Upon arrival at the laboratory, fish specimens were measured, weighed, and dissected to isolate muscle, liver, and gill tissues, which were then processed for heavy metal analysis. The average size of the *Nemipterus japonicus, Oreochromis mossambicus* and *Lates niloticus* fish were recorded as 15.9 ± 0.37, 17.01 ± 0.18, and 15.8 ± 0.45 cm, respectively. These organs were then subjected to dehydration in a hot-air oven at 40–50°C until achieved a constant weight. The samples that had been completely dried were then grounded and homogenized for further processing with a mortar and pestle.

### 2.3 Sample preparation

Heavy metals were extracted using an acid digestion method (24). A 25 mg portion of the dried sample was digested in 8 mL of NICE Lab grade 65% nitric acid (HNO_3_) and 1 mL of Merck 30% hydrogen peroxide (H_2_O_2_) using a closed heat-resistant vessel (23). Digestion was carried out at 220°C for 8 h on a hot plate. The resulting mineralized solution was transferred to a 10 mL volumetric flask, diluted with 2% HNO_3_, and subjected to 10-fold dilution for Q-ICP-MS analysis (23). Calibration standards were prepared from multi-element stock solutions and run in triplicate to establish calibration curves. The calibration range for As, Cd, Cr, Pb, Sr, V is 0.01–100 μg/L and for Hg: 0.005–50 μg/L. All calibration curves exhibited correlation coefficients (R^2^) greater than 0.999, indicating excellent linearity.

### 2.4 Instrumental set-up for analysis

Heavy metal concentrations (As, Cd, Cr, Pb, Sr, V and Hg) were determined using Inductively Coupled Plasma Mass Spectrometry (ICP-MS) (Perkin Elmer NexION 1000) at Vellore Institute of Technology, India. This facility is supported by the Department of Science and Technology, New Delhi, under the “Promotion of University Research and Scientific Excellence (PURSE)” program. To minimize contamination, a triple cone interface system and a thermally controlled spray chamber were used. The RF forward power was set at 1600 W, and analyses were conducted in Helium Kinetic Energy Discrimination (KED) mode without collision cell technology (23).

### 2.5 Quality control and assurance

Blanks and spiked matrix samples were analyzed with every batch (1 blank and 1 spiked sample for every 10 tissue samples). LOD ranged between 0.001 and 0.010 μg/kg, and LOQ between 0.005 and 0.030 μg/kg depending on the element. Analytical precision was confirmed by running each sample in triplicate, with a relative standard deviation (RSD) of <5% for all measurements.

### 2.6 Health risk assessment

Key equations used for assessing heavy metal exposure risks in human health are given in Table 1. These were calculated using standard equations established by the United States Environmental Protection Agency (USEPA) and the European Food Safety Authority (EFSA). These calculations are based on established models from the United States Environmental Protection Agency (USEPA) and the European Food Safety Authority (EFSA), estimate daily and weekly intake levels, evaluate potential health risks, and determine safe consumption limits. Here, the ingestion rate (IR) was 75 g/day for children and 150 g/day for adults, with body weight (BW) set at 70 kg for adults and 20 kg for children (25). PTWI values were sourced from EFSA (26), while RfD values were taken from FAO/WHO (5) and WHO (27). Exposure frequency (EFr) was 365 days/year, and exposure duration (ED) was 70 years (28). CSF values for Cd, Pb, Cr, and As were derived from FAO/WHO (5) and Gao et al. (29).

**Table 1:** Risk assessment equations for heavy metal exposure.

**Equation**	**Formula**	**Description**	**Parameters**	**Reference**
Estimated Daily Intake (EDI)	EDI = (Mc × IR)/BW	Daily intake of metal per kg body weight.	MC = Metal concentration (mg/kg), IR = Ingestion rate (kg/day), BW = Body weight (kg).	(25, 53)
Estimated Weekly Intake (EWI)	EWI= [(Mc × IR)/BW] × 7	Total weekly exposure to metal.	MC = Metal concentration in food (mg/kg), IR = Ingestion rate (kg/day), BW = Body weight (kg).	(5, 25, 53)
Maximum Daily Intake (MDI)	$MDI=\;\frac{\left(PTWI\times Body\;Weight\right)}{7}$	Safe daily limit based on PTWI.	PTWI = Provisional tolerable weekly intake (mg/kg/week) BW = Body weight (kg).	(27)
Daily Intake Limit (DIL)	$DIL=\frac{\;RfD\;\times BW}{C}$	Max safe food quantity consumed per day.	RfD = Reference dose (mg/kg/day), BW = Body weight (kg), C = Metal concentration in food (mg/kg).	(5)
Maximum Acceptable Daily Intake (MADI/CRlim)	$CRlim\;or\;MADI=\frac{RfD\;or\;RfC\times BW}{C}$	Acceptable upper daily intake without harm.	RfD = Reference dose (mg/kg/day), BW = Body weight (kg), C = Metal concentration in food (mg/kg).	(5)
Target Hazard Quotient (THQ)	$THQ=\;\frac{EFr\times ED\times IR\times C}{RfD\;\times BW\;\times AT}$	Evaluates non-carcinogenic health risk (THQ > 1 implies potential risk).	EFr = Exposure frequency (days/year), ED = Exposure duration (years), IR = Ingestion rate (g/day), C = Contaminant concentration (mg/kg), RfD = Reference dose (mg/kg/day), BW = Body weight (kg), AT = Averaging time (days).	(28)
Cancer Risk (CR)	*CR* = *EDI*×*CSF*	Lifetime probability of cancer from metal exposure.	EDI = Estimated daily intake (mg/kg/day), CSF = Cancer slope factor (mg/kg/day^)−1^.	(25, 29, 53)

## 3 Results and discussion

### 3.1 Trace metal accumulation in fish tissues

The results of this study revealed varying concentrations of trace metals (As, Cd, Cr, Hg, Pb, Sr, and V) across three fish species (*Nemipterus japonicus, Oreochromis mossambicus, and Lates niloticus*) and tissues (liver, gills, and muscle) (Table 2). The concentrations ranged from 0.044 to 0.096 μg/kg for As, 0.696–0.778 μg/kg for Cd, 5.259–12.399 μg/kg for Cr, 0.020–0.066 μg/kg for Hg, 15.400–17.649 μg/kg for Pb, 1.068–15.200 μg/kg for Sr, and 0.150–1.208 μg/kg for V.

**Table 2:** Heavy metal concentration in various organs (liver, gills, and muscle) of *Nemipterus japonicus, Oreochromis mossambicus* and *Lates calcarifer* collected from Chennai, TN (in μg kg^−1^) (values represented in mean ± standard deviation; *n* = 6) (54, 55).

**Fish**	**Organs**	**As**	**Cd**	**Cr**	**Hg**	**Pb**	**Sr**	**V**
*Nemipterus japonicus*	Liver	0.034 ± 0.004	0.731 ± 0.035	5.846 ± 1.292	0.020 ± 0.004	15.829 ± 0.854	1.195 ± 0.641	0.314 ± 0.234
	Gills	0.096 ± 0.102	0.697 ± 0.008	5.284 ± 1.283	0.021 ± 0.004	15.400 ± 0.415	2.430 ± 2.488	0.157 ± 0.018
	Muscle	0.308 ± 0.083	0.698 ± 0.015	6.611 ± 0.661	0.027 ± 0.004	15.524 ± 0.087	3.711 ± 0.955	0.169 ± 0.007
*Oreochromis mossambicus*	Liver	0.121 ± 0.010	0.731 ± 0.031	8.388 ± 0.250	0.050 ± 0.010	16.766 ± 0.718	14.135 ± 0.839	0.482 ± 0.016
	Gills	0.091 ± 0.026	0.778 ± 0.075	6.831 ± 1.501	0.066 ± 0.060	16.894 ± 1.467	3.302 ± 1.324	0.484 ± 0.251
	Muscle	0.218 ± 0.218	0.748 ± 0.066	12.399 ± 5.384	0.048 ± 0.040	17.649 ± 1.802	9.623 ± 3.496	1.208 ± 1.371
*Lates niloticus*	Liver	0.443 ± 0.079	0.777 ± 0.021	8.330 ± 0.792	0.031 ± 0.005	17.250 ± 0.698	3.199 ± 0.870	0.227 ± 0.022
	Gills	0.315 ± 0.252	0.718 ± 0.025	7.114 ± 1.300	0.038 ± 0.015	15.912 ± 0.560	15.200 ± 12.859	0.318 ± 0.142
	Muscle	0.141 ± 0.202	0.696 ± 0.009	5.259 ± 1.682	0.024 ± 0.002	15.364 ± 0.155	1.068 ± 0.843	0.150 ± 0.024

In *Nemipterus japonicus*, the liver exhibited the highest recorded lead (Pb) concentration at 15.829 ± 0.854 μg/kg, supporting findings that Pb preferentially accumulates in hepatic tissues due to its affinity for sulfhydryl (-SH) groups in hepatic enzymes, facilitating bioaccumulation (30). Previous studies have similarly reported that Pb binds to metallothioneins and sulfur-containing proteins in the liver, enhancing its retention in this organ (31, 32). Muscle tissues revealed an elevated concentration of chromium (Cr) at 6.611 ± 0.661 μg/kg, whereas mercury (Hg) levels remained consistently low across all examined tissues. This finding aligns with study conducted by Shah et al. (33) on *Ctenopharyngodon idella*, where muscle tissues accumulated higher chromium concentrations than gill tissues, highlighting muscle as a significant reservoir for Cr accumulation (33). Similarly, while mercury generally accumulates in fish muscle, the low Hg levels observed in *N. japonicus* may indicate species-specific detoxification mechanisms or lower environmental exposure. These findings highlight the importance of hepatic and muscular tissues as vital reservoirs for the accumulation of particular toxic metals in this species. The ranking of contamination levels across different tissues is established as follows, from the highest concentration to the lowest: in liver tissue, it is Pb > Cr > Cd > Sr > V > As > Hg, and for both muscle tissues and gills, the order is identical, which is Pb > Cr > Sr > Cd > As > V > Hg. This clearly indicates that lead (Pb) is the foremost contaminant present in all organs of this species.

In *Oreochromis mossambicus*, muscle tissues show the most considerable amounts of Pb (17.649 ± 1.802), Cr (12.399 ± 5.384), As (0.218 ± 0.218), and V (1.208 ± 1.371), which brings forth important toxicity issues related to eating this fish. On the other hand, Sr (14.135 ± 0.839) revealed the maximum levels within the hepatic tissues, while Cd (0.778 ± 0.075) and Hg (0.066 ± 0.060) were detected at significantly increased levels in the gills of this species. The concentrations of Hg were minimal across all tissues, suggesting a low degree of mercury contamination within the study area. Corresponding studies indicate that *Oreochromis mossambicus* possesses an increased cumulative risk, particularly concerning the health and safety of pediatric populations (34, 35). In all three tissues, consistent contamination levels were noted, ranked from the highest concentration down to the lowest: Pb > Cr > Sr > Cd > V > As > Hg. A 2014 study in India's Subarnarekha River indicated elevated arsenic levels, while varying amounts of cadmium and lead were noted across different fish species (36). A related investigation conducted in 2021 in the Gulf of Guinea also reported arsenic levels of 8.46 ± 2.42 μg/g in *Penaeus notialis* (37).

Regarding *Lates niloticus*, the hepatic organ displayed the most elevated concentrations of Pb (17.250 ± 0.698) and Cd (0.777 ± 0.032), thereby emphasizing its role as the central organ for metal accumulation in this particular species. Studies indicate that cadmium pollution, connected to several harmful effects on both humans and other animal species, is mostly related to reproductive issues and developmental irregularities (38–41). The gills demonstrate considerable quantities of Sr (15.200 ± 12.859) in relation to water metal exposures, suggesting a selective bioaccumulation phenomenon within this tissue. Additionally, the contamination levels found in each of the three tissues consistently align, presented in this order from highest to lowest concentration: in hepatic tissues, the order is Pb > Cr > Sr > Cd > As > V > Hg, and for gills and muscle tissues, it mirrors this same ranking of Pb > Cr > Sr > Cd > V > As > Hg.

Overall, the result indicate that the liver serves as a major site for metal accumulation, particularly for Pb, Cd, and Cr, due to its detoxification function. Consistently elevated Pb levels in all species are concerning given its well-known toxic effects on biological systems. Similar findings reported by 10 also highlighted Pb-induced hepatotoxicity, developmental delays, and behavioral disorders (12). Although muscle tissues generally contained lower concentrations, they still warrant attention due to their significance in human diets.

### 3.2 Health risk assessment

The estimated daily intake (EDI) values (Table 3) provide insights into potential health risks from consuming these contaminated fish. While some metals posed minimal risks, others, particularly Pb and Cd, raised significant concerns, especially for children and frequent consumers.

**Table 3:** Summary of estimated daily intake (EDI), estimated weekly intake (EWI), daily intake level (DIL), and maximum acceptable daily intake (CRlim) values for heavy metals in fish samples.

**Fish Species**		**Estimated Daily Intake (EDI) in** μ**g/kg/day**		**Estimated Weekly Intake (EWI) in** μ**g/kg/day**		**Daily Intake Limit for fish in Kg per day**		**CRlim/Maximum acceptable Daily Intake (Kg/day)**	
	**HMs**	**Children**	**Adult**	**Children**	**Adult**	**Children**	**Adult**	**Children**	**Adult**
*Nemipterus japonicus*	As	1.155	0.660	8.085	4.620	19.480	68.181	19.480	68.181
	Cd	2.617	1.495	18.3225	10.470	28.653	100.286	28.653	100.286
	Cr	24.791	14.166	173.538	99.165	4,537.891	15,882.619	4,537.891	15,882.619
	Hg	0.101	0.057	0.708	0.405	74.074	259.259	74.074	259.259
	Pb	58.215	33.265	407.505	232.860	0.386	1.352	0.386	1.352
	Sr	13.916	7.952	97.413	55.665	18.862	66.019	18.862	66.019
	V	0.633	0.362	4.436	2.535	1,065.088	3,727.810	1,065.088	3,727.810
*Oreochromis mossambicus*	As	0.817	0.467	5.722	3.270	27.522	96.330	27.522	96.330
	Cd	2.805	1.602	19.635	11.220	26.737	93.582	26.737	93.582
	Cr	46.496	26.569	325.473	185.985	2,419.549	8,468.424	2,419.549	8,468.424
	Hg	0.180	0.102	1.260	0.720	41.666	145.833	41.666	145.833
	Pb	66.183	37.819	463.2862	264.735	0.339	1.189	0.339	1.18
	Sr	36.086	20.620	252.603	144.345	7.274	25.459	7.274	25.459
	V	4.530	2.588	31.701	18.120	149.006	521.523	149.006	521.523
*Lates niloticus*	As	0.528	0.302	3.701	2.115	42.553	148.936	42.553	148.936
	Cd	2.610	1.491	18.270	10.440	28.735	100.574	28.735	100.574
	Cr	19.721	11.269	138.048	78.885	5,704.506	19,965.772960	5,704.506	19,965.772
	Hg	0.090	0.051	0.630	0.360	83.333	291.666	83.3333	291.666
	Pb	57.615	32.922	403.305	230.460	0.390	1.366	0.390	1.366
	Sr	4.005	2.288	28.035	16.020	65.543	229.400	65.543	229.400
	V	0.562	0.321	3.937	2.250	1,200.000	4,200.000	1,200.000	4,200.000

*RfD (Reference Dose) values used for risk assessment (in mg/kg/day):* Arsenic (As) = 0.0003, Cadmium (Cd) = 0.001, Chromium (Cr(III)) = 1.5, Chromium (Cr(VI)) = 0.003, Mercury (Hg) = 0.0001, Lead (Pb) = 0.0003, Vanadium (V) = 0.0035.

Sources: (54, 55).

EDI values for arsenic ranged from 0.530 to 1.160 μg/kg/day across the tested species, suggesting a relatively low risk of acute toxicity. However, chronic exposure remains a concern due to As's potential carcinogenic effects, especially for vulnerable groups like children (42). A study assessing dietary arsenic intake in Japan found a positive association between inorganic arsenic exposure and an increased risk of lung and kidney cancers, particularly in men (43). Similarly, a study evaluated the cancer risk associated with inorganic arsenic exposure from consuming tilapia in areas hyperendemic for Blackfoot Disease in Taiwan (44). The research highlighted elevated health risks due to inorganic arsenic exposure through fish consumption. Cadmium levels varied from 2.610 to 2.810 μg/kg/day, suggesting moderate contamination. Given Cadmium's cumulative toxicity, prolonged exposure could have severe health implications, particularly affecting kidney function over time (45). In case of Chromium, it exhibited a broad EDI range of 19.720–46.500 μg/kg/day, with the highest accumulation observed in *Oreochromis mossambicus* species (Table 3). While Cr is an essential trace element, excessive intake in the form of Cr(VI) poses serious carcinogenic and systemic toxicity risks (46). The mercury intake was notably low, with values ranging from 0.090 to 0.180 μg/kg/day, suggesting minimal contamination. Although it is recognized as a prevalent environmental pollutant, in larger levels it can induce a multitude of detrimental impacts on human health, encompassing dysfunction of the nervous system and disorders related to development (47). Mercury intake was notably low (0.090–0.180 μg/kg/day), indicating minimal contamination. However, given Mercury's neurotoxic properties, continuous monitoring is necessary, particularly for populations with high fish consumption (46).

Lead exposure was found relatively higher (57.620–66.180 μg/kg/day) than similar studies, and may pose severe risks, especially to children's neurological and cognitive development. Few studies have linked Pb in fish to developmental disorders. Wherein, strontium values varied between 4.010 and 36.090 μg/kg/day, showing noticeable interspecies differences in accumulation. Though Sr is generally less toxic, excessive intake may contribute to cumulative skeletal toxicity. Vanadium levels were among the lowest, ranging from 0.560 to 4.530 μg/kg/day, indicating minimal immediate risk, though prolonged exposure could still have adverse health effects (48).

The Daily Intake Limit (DIL) values reflect the maximum safe fish consumption levels before exceeding toxic thresholds. The DIL for As ranged from 19.480 to 42.550 kg/day, reinforcing its relatively low risk at the detected concentrations. However, Cd's DIL values of 26.730–28.740 kg/day indicate a moderate health risk, especially for frequent consumers. Cr (VI) contamination exhibited the highest DIL values, ranging from 2,419.550 to 5,704.510 kg/day, confirming negligible toxicity risks unless fish consumption reaches impractically high levels. Hg showed a notably low DIL of 74.070–83.330 kg/day, underscoring its significant toxic potential. Similarly, Pb presented the most immediate risk, with a DIL range of 0.340–0.390 kg/day, suggesting that even minor fish consumption could lead to hazardous Pb exposure. DIL values of Sr spanned 7.270–65.540 kg/day, reflecting variable but generally moderate toxicity concerns. In overall, V with the highest DIL range of 1,065.080–1,200.000 kg/day, exhibited the lowest toxicity risk among the analyzed metals. While metals such as Cr and Sr also appear less hazardous at current levels, the elevated Pb and Cd concentrations demand urgent regulatory oversight. Hg contamination, although relatively low, still warrants caution due to its neurotoxic nature. Previous research, including studies by Mahaffey (49), has demonstrated that even low Hg concentrations in fish contribute to neurodevelopmental issues in children, reinforcing the importance of consumption advisories, particularly for vulnerable populations. Given the cumulative effects of these metals, long-term exposure assessments and dietary restrictions for at-risk populations, such as children and pregnant women, are strongly recommended.

Table 4 delineates the acceptable daily and weekly consumption thresholds for noted heavy metals applicable to both children and adults. Certain consumption limits function as essential standards to assess the possible health hazards linked to dietary exposure to certain hazardous substances. The maximum daily intake (MDI) for Arsenic is 42.860 mg/day for children and 150 mg/day for adults, with a maximum weekly intake (MWI) of 300 mg/week for children and 1,050 mg/week for adults. Arsenic is a recognized carcinogen, and prolonged exposure, even at minimal concentrations, can present considerable health hazards, underscoring the need of its control.

**Table 4:** MDI and MWI [PTWI for arsenic (inorganic), Cd, Pb, Hg are 15, 7, 25, 4 μg/kg body weight per week (55).

**Heavy metals**	**Maximum Daily Intake (in mg/day)**		**Maximum Weekly Intake (mg/week)**	
	**Children**	**Adult**	**Children**	**Adult**
As	42.857	150.000	300	1050
Cd	20.000	70.000	140	490
Cr	71.429	250.000	500	1750
Hg	11.429	40.000	80	280
Pb	71.429	250.000	500	1750
Sr	20000.000	70000.000	140000	490000
V	20.000	70.000	140	490

Cadmium demonstrated MDI values of 20 mg/day for children and 70 mg/day for adults, with MWI values of 140 mg/week for children and 490 mg/week for adults. Cadmium is a cumulative toxin that predominantly impacts renal function and skeletal health; hence, even mild exposure over time may result in significant health issues.

Chromium, a vital trace element, exhibited MDI values of 71.430 mg/day for children and 250 mg/day for adults, with MWI values of 500 mg/week for children and 1,750 mg/week for adults. Although chromium is advantageous in trace quantities, excessive exposure, especially to its poisonous Cr (VI) variant, raises concerns regarding carcinogenicity and systemic toxicity.

Mercury levels were recorded at 11.430 mg/day for children and 40 mg/day for adults, with maximum weekly intake (MWI) values of 80 mg/week for children and 280 mg/week for adults. Due to Hg's neurotoxic properties, especially in young children and pregnant women, dietary intake must be meticulously regulated.

Lead, a very hazardous heavy metal, with MDI values of 71.430 mg/day for children and 250 mg/day for adults, while the MWI values were 500 mg/week for children and 1,750 mg/week for adults. A study by Kumar et al. (50) at Ennore Creek, Tamil Nadu, India, reported significantly high lead contamination levels in various fish species, which aligns with our findings. For example, lead concentrations in *Penaeus monodon, Perna viridis, Crossosstrea madrasensis*, and *Mugil cephalus* ranged from 2.590 to 4.370 μg/g (50), indicating substantial contamination. Given that even low Pb exposure can cause severe neurodevelopmental damage, its presence in dietary sources is particularly alarming.

Strontium displayed much higher allowed intake levels, with MDI values of 20,000 mg/day for children and 70,000 mg/day for adults, with MWI values of 140,000 mg/week for children and 490,000 mg/week for adults. While Sr is regarded less hazardous than other metals, excessive buildup may contribute to bone health concerns over time.

Vanadium revealed MDI values of 20 mg/day for children and 70 mg/day for adults, with MWI values of 140 mg/week for children and 490 mg/week for adults. Although V toxicity is very minimal at these levels, extended exposure nevertheless necessitates monitoring because to its potential impacts on metabolic and cardiovascular health.

The findings underline the important necessity for regular monitoring of heavy metal exposure in dietary sources. Even at low concentrations, As, Cd, Hg, and Pb pose significant cumulative toxicity risks. Despite higher allowable limits, Sr and V may still pose long-term health risks. Given these hazards, regulatory agencies must implement strong safety measures to guarantee that heavy metal concentrations in food sources remain substantially below the set standards, particularly for vulnerable groups like children.

Table 5 thoroughly evaluates heavy metal exposure through the consumption of three fish species: *Nemipterus japonicus, Oreochromis mossambicus*, and *Lates niloticus*. This evaluation contains critical metrics such as Target Hazard Quotient (THQ), Hazard Index (HI), and Cancer Risk (CR) for both children and adults. The Target Hazard Quotient (THQ) serves as a critical metric for risk assessment, employed to ascertain the possible health risks linked to prolonged exposure to a chemical contaminant via dietary consumption. A THQ value that falls below 1 signifies that the exposure level is improbable to induce detrimental health effects, whereas a THQ exceeding 1 indicates the likelihood of potential health hazards (11, 51, 52). The statistics suggest that the hazard of non-carcinogenic repercussions from swallowing these fish species is insignificant, given all THQ values were judged to be <1. This suggests that at typical intake rates, these fish are usually safe for ingestion (37, 51).

**Table 5:** Calculated values of THQ, HI, and CR (“–” represents not determined) (12).

**Fish Species**		**Target Hazard Quotient (THQ)**		**Hazard Index**		**Cancer Risk**	
	**HMs**	**Children**	**Adult**	**Children**	**Adult**	**Children**	**Adult**
*Nemipterus japonicus*	As	0.003	0.002	0.205	0.117	1.73 × 10^−6^	9.90 × 10^−7^
	Cd	0.002	0.001			1.31 × 10^−10^	7.47 × 10^−11^
	Cr	0.000	0.000			1.02 × 10^−6^	5.82 × 10^−7^
	Hg	0.001	0.001			_	_
	Pb	0.194	0.110			4.95 × 10^−7^	2.83 × 10^−7^
	Sr	0.004	0.002			_	_
	V	0.000	0.000			_	_
*Oreochromis mossambicus*	As	0.003	0.002	0.239	0.136	1.23 × 10^−6^	7.01 × 10^−7^
	Cd	0.003	0.002			1.40 × 10^−10^	8.01 × 10^−11^
	Cr	0.000	0.000			1.91 × 10^−6^	1.09 × 10^−6^
	Hg	0.002	0.001			_	_
	Pb	0.221	0.126			5.63 × 10^−7^	3.21 × 10^−7^
	Sr	0.010	0.006			_	_
	V	0.001	0.000			_	_
*Lates niloticus*	As	0.002	0.001	0.198	0.113	7.93 × 10^−7^	4.53 × 10^−7^
	Cd	0.003	0.001			1.31 × 10^−10^	7.40 × 10^−11^
	Cr	0.000	0.000			8.08 × 10^−7^	4.62 × 10^−7^
	Hg	0.001	0.001			_	_
	Pb	0.192	0.109			4.91 × 10^−7^	2.81 × 10^−7^
	Sr	0.001	0.001			_	_
	V	0.000	0.000			_	_

Cancer risk (CR) assessment examines the probability of developing cancer over a lifetime due to exposure to carcinogenic agents. Arsenic (As) presented the highest concern, with CR values for *Nemipterus japonicus* ranging from 1.7325 × 10^−6^ (children) to 0.99 × 10^−6^ (adults). Although these values are near the safety threshold of 1 × 10^−6^, they remain within acceptable limits for public health.

Similar patterns were observed in *Oreochromis mossambicus* and *Lates niloticus*, indicating a borderline cancer risk, particularly for children. Cadmium (Cd) levels were very low across all species, with CR as high as 1.31 × 10^−10^ for children in *Nemipterus japonicus*. These values are negligible, suggesting little cancer risk from cadmium exposure from fish eating.

However, chromium (Cr) levels were more concerning, especially in *Nemipterus japonicus*, where the CR value for children reached 1.02 × 10^−6^. It is essential to note that this value represents total chromium without differentiating between Cr (III), which is an essential nutrient, and Cr (VI), which is highly carcinogenic. Further research is needed to determine the exact contribution of Cr (VI) to the overall risk. Lead (Pb) levels were also examined, and CR values staying below the acceptable threshold of 1 × 10^−6^. For instance, *Nemipterus japonicus* showed CR values of 04.95 × 10^−7^ for newborns and 2.83 × 10^−7^ for adults. While these statistics reflect a modest cancer risk, it is crucial to recognize that lead is largely connected with non-carcinogenic developmental repercussions, especially in children, even at trace levels.

### 3.3 Limitations

One of the primary limitations of this study is the relatively small sample size (*n* = 18), with six individuals fishes analyzed within a species. While this provided valuable preliminary data on organ-specific and species specific heavy metal accumulation, a larger sample pool would be necessary to increase statistical robustness and improve the representativeness of the results. Future investigations are recommended to involve seasonal sampling across multiple locations and a higher number of biological replicates to strengthen the ecological and public health implications.

## 4 Conclusion

Based on the observed data, the concentrations of heavy metals, including Arsenic (As), Cadmium (Cd), Chromium (Cr), Mercury (Hg), Lead (Pb), Strontium (Sr), and Vanadium (V), varied across the three fish samples, with ranges of 0.096–0.044 μg/kg, 0.696–0.778 μg/kg, 5.259–12.399 μg/kg, 0.020–0.660 μg/kg, 15.400–17.649 μg/kg, 1.068–15.200 μg/kg, and 0.150–1.208 μg/kg, respectively. Among the samples, the muscle tissues of *Oreochromis mossambicus* exhibited the highest levels of heavy metal contamination, primarily due to its elevated chromium (Cr) concentration of 12.399 μg/kg. The Hazard Index (HI) analysis revealed that *Oreochromis mossambicus* posed the highest potential risk, with HI values of 0.238 for children and 0.136 for adults. Despite this, the Target Hazard Quotient (THQ) for all metals remained below 1, indicating that the fish samples are safe for human consumption in terms of heavy metal contamination. This suggests that while *Oreochromis mossambicus* has relatively higher contamination levels among the selected species, it does not pose a significant health risk and can be considered safe for consumption within the observed concentration ranges. Furthermore, it is recommended to clean the fish before cooking and one must follow a proper cooking method to avoid any existing contamination in food. Given these findings, routine monitoring of heavy metals in aquatic food sources remains essential. Continuous surveillance not only supports consumer safety but also contributes to sustainable environmental and public health management strategies.

## Data Availability

The original contributions presented in the study are included in the article/supplementary material, further inquiries can be directed to the corresponding author.
