# Correction: ICP-MS-based quantitative analysis and risk assessment of metal(loid)s in fish species from Chennai, India

**DOI:** 10.3389/fpubh.2025.1697328

**Published:** 2025-11-19

**Authors:** Suryapratap Ray, Gracy Anu Francis, Sumit Sudhir Pathak, Pooja Chavan, Rahul Vashishth

**Affiliations:** 1 Department of Biosciences, School of Biosciences and Technology, Vellore Institute of Technology, Vellore, India; 2 Division of Food Processing Technology, Karunya Institute of Technology and Sciences, Coimbatore, Tamil Nadu, India; 3 VIT School of Agricultural Innovation and Advanced Learning, Vellore Institute of Technology, Vellore, India

**Keywords:** trace elements, environmental impacts, health risk assessment, sea food, contaminated fish

Author Chitra Jangid was erroneously included as an author in the published article. The correct author list reads:

Suryapratap Ray^1^, Gracy Anu Francis^1^, Sumit Sudhir Pathak^2^, Pooja Chavan^3^ and Rahul Vashishth^1*^

^1^Department of Biosciences, School of Biosciences and Technology, Vellore Institute of Technology, Vellore, India

^2^Division of Food Processing Technology, Karunya Institute of Technology and Sciences, Coimbatore, Tamil Nadu, India

^3^VIT School of Agricultural Innovation and Advanced Learning, Vellore Institute of Technology, Vellore, India

The **Author contributions** statement has been corrected to read:

SR: Conceptualization, Writing – original draft, Methodology, Investigation, Resources, Formal analysis, Visualization. GF: Validation, Writing – original draft, Visualization. SP: Data curation, Resources, Writing – original draft, Formal analysis. PC: Writing – original draft, Resources, Validation, Visualization. RV: Supervision, Writing – original draft, Writing – review & editing, Data curation, Conceptualization.

The **Acknowledgment** statement has been amended to read as follows:

“The authors are grateful to the Department of Science and Technology, New Delhi, India for providing financial support to acquire “Inductively Coupled Plasma Mass Spectrometry (ICP-MS)” through “Promotion of University Research and Scientific Excellence (PURSE)” under Grant No. SR/PURSE/2020/34 (TPN 56960) and carry out the work. Authors acknowledge Chitra Jangid for her valuable assistance in manuscript correction and editing. Her expertise and constructive inputs enhanced the quality of work.”

The original version of this article has been updated.

